# Supplementary material for: Saturation genome editing maps the functional spectrum of pathogenic VHL alleles
Source: Nat Genet. 2024 Jul 5;56(7):1446–55. doi: 10.1038/s41588-024-01800-z (PMC11250436; doi:10.1038/s41588-024-01800-z)
Supplement: Supplementary file 1 — Supplementary Note. [file 41588_2024_1800_MOESM1_ESM.pdf]

nature genetics

Supplementary information https://doi.org/10.1038/s41588-024-01800-z

Saturation genome editing maps the functional spectrum of pathogenic *VHL* alleles

In the format provided by the authors and unedited

Table of Contents

2 … Supplementary Note 1. Optimizing the SGE protocol to assay *VHL*.

2 … Supplementary References

Supplementary Tables provided separately:

Supplementary Table 1. SGE function scores and RNA scores for 2,268 *VHL* SNVs.

Supplementary Table 2. SGE data for variants with established clinical phenotypes.

Supplementary Table 3. SGE data for SNVs with recessive phenotypes and clinically relevant SNVs assayed in intron 1.

Supplementary Table 4. Oligonucleotides used to perform SGE of *VHL*.

Supplementary Note 1. Optimizing the SGE protocol to assay *VHL*.

We substantially optimized SGE to enable accurate measurement of functional effects of *VHL* SNVs. Sequencing of initial libraries revealed two regions of exon 1 to have skewed variant distributions at sites of repetitive, GC-rich sequence (**Extended Data Fig. 2a-e**). Additional synonymous mutations were therefore engineered in these regions, resulting in improved library uniformity (**Extended Data Fig. 2f-l**).

For four exonic regions initially assayed using normal HAP1 culture media, only modest growth defects were observed for expected LoF variants (**Fig. 1e, Extended Data Fig. 3**). HAP1 cells can revert to diploidy with prolonged culture[64], a phenomenon that could weaken recessive effects measured in multiplex. Recently, 10-deacetyl-baccatin-III (DAB) was identified via small molecule screening to select for haploid cells[32]. Therefore, we next performed SGE for all *VHL* regions in media containing 2.5 μM DAB. This led to a substantial improvement in dynamic range (**Fig. 1f, Extended Data Fig. 3**). In exon 2, for example, the median function score of nonsense and canonical splice site SNVs dropped 4-fold, from -0.62 to -2.49. Across all SGE regions assayed with and without DAB, there were 39.3% more significantly depleted SNVs identified in DAB-treated cells. Therefore, we used only data from SGE experiments performed with DAB to calculate final function scores.

Supplementary References

64. Beigl, T. B., Kjosås, I., Seljeseth, E., Glomnes, N. & Aksnes, H. Efficient and crucial quality control of HAP1 cell ploidy status. *Biol. Open* **9**, (2020).
