# Supplementary material for: Saturation genome editing maps the functional spectrum of pathogenic VHL alleles
Source: Nat Genet. 2024 Jul 5;56(7):1446–55. doi: 10.1038/s41588-024-01800-z (PMC11250436; doi:10.1038/s41588-024-01800-z)
Supplement: Supplementary file 2 — Reporting Summary [file 41588_2024_1800_MOESM2_ESM.pdf]

nature portfolio

Corresponding author(s): Gregory M. Findlay

Last updated by author(s): 4/25/2024

# Reporting Summary

Nature Portfolio wishes to improve the reproducibility of the work that we publish. This form provides structure for consistency and transparency in reporting. For further information on Nature Portfolio policies, see our Editorial Policies and the Editorial Policy Checklist.

## Statistics

For all statistical analyses, confirm that the following items are present in the figure legend, table legend, main text, or Methods section.

| n/a | Confirmed | |
|---|---|---|
| ☐ | ☒ | The exact sample size (*n*) for each experimental group/condition, given as a discrete number and unit of measurement |
| ☐ | ☒ | A statement on whether measurements were taken from distinct samples or whether the same sample was measured repeatedly |
| ☐ | ☒ | The statistical test(s) used AND whether they are one- or two-sided<br>*Only common tests should be described solely by name; describe more complex techniques in the Methods section.* |
| ☐ | ☒ | A description of all covariates tested |
| ☐ | ☒ | A description of any assumptions or corrections, such as tests of normality and adjustment for multiple comparisons |
| ☐ | ☒ | A full description of the statistical parameters including central tendency (e.g. means) or other basic estimates (e.g. regression coefficient) AND variation (e.g. standard deviation) or associated estimates of uncertainty (e.g. confidence intervals) |
| ☐ | ☒ | For null hypothesis testing, the test statistic (e.g. *F*, *t*, *r*) with confidence intervals, effect sizes, degrees of freedom and *P* value noted<br>*Give P values as exact values whenever suitable.* |
| ☒ | ☐ | For Bayesian analysis, information on the choice of priors and Markov chain Monte Carlo settings |
| ☒ | ☐ | For hierarchical and complex designs, identification of the appropriate level for tests and full reporting of outcomes |
| ☐ | ☒ | Estimates of effect sizes (e.g. Cohen's *d*, Pearson's *r*), indicating how they were calculated |

*Our web collection on statistics for biologists contains articles on many of the points above.*

## Software and code

Policy information about availability of computer code

| | |
|---|---|
| Data collection | Illumina sequencing .bcl files were processed to .fastq files using bcl2fastq2 (v2.17.1.14). |
| Data analysis | Analysis of sequencing data was performed as fully described in Methods, using a custom pipeline to process .fastq files to variant-level function scores and RNA scores. This pipeline uses SeqPrep v1.3.2 for read merging, needleall (EMBOSS v6.6.0.0) for sequence alignment, and custom scripts written in Python v2.7.5. Subsequent statistical analysis was performed in R v3.6.3 using RStudio v1.4.1106. Micrographs were analyzed using Fiji ImageJ2 v2.14.0 and flow cytometry data were analyzed with FlowJo v10.10. FoldX v5.0 and PyMol v.2.5.4 were used for structural analysis. GraphPad Prism v10.1.2 was used to analyze age-related ccRCC penetrance.<br><br>Code used in this study is available on GitHub (https://github.com/TheGenomeLab/VHL-SGE) and has been archived to Zenodo (https://zenodo.org/records/11065771) |

For manuscripts utilizing custom algorithms or software that are central to the research but not yet described in published literature, software must be made available to editors and reviewers. We strongly encourage code deposition in a community repository (e.g. GitHub). See the Nature Portfolio guidelines for submitting code & software for further information.

# Data

Policy information about availability of data

All manuscripts must include a data availability statement. This statement should provide the following information, where applicable:

- Accession codes, unique identifiers, or web links for publicly available datasets
- A description of any restrictions on data availability
- For clinical datasets or third party data, please ensure that the statement adheres to our policy

All function scores and RNA scores are included in Supplementary Table 1, as well as NGS read counts. Function scores are also available for visualization at https://vhl-board.onrender.com/ and have been deposited to MAVE-DB (urn:mavedb:00000675-a). Fastq files are publicly available (European Nucleotide Archive accession: PRJEB75229). Unprocessed western blots are included as Source Data.

Structural data (PDB: 1LM8) was accessed from the Protein Data Bank (https://www.rcsb.org/structure/1lm8). ClinVar, cBioPortal, and VHLdb data are available via https://www.ncbi.nlm.nih.gov/clinvar/, https://www.cbioportal.org/, and http://vhldb.bio.unipd.it/, respectively. UK Biobank, TOPMed, and gnomAD data are accessible via https://app.genebass.org/, https://bravo.sph.umich.edu/freeze8/hg38/, and https://gnomad.broadinstitute.org/, respectively. CADD scores can be found at https://cadd.gs.washington.edu/download, and missense variant scores from REVEL, boostDM, EVE, and VARITY are available at https://sites.google.com/site/revelgenomics/downloads, https://www.intogen.org/boostdm/search?ttype=RCCC&gene=VHL, https://evemodel.org/, and http://varity.varianteffect.org/, respectively.

# Research involving human participants, their data, or biological material

Policy information about studies with human participants or human data. See also policy information about sex, gender (identity/presentation), and sexual orientation and race, ethnicity and racism.

| | |
|---|---|
| Reporting on sex and gender | The Freiburg VHL Registry includes patients screened at least once until 2023 at the von Hippel-Lindau Outpatient Clinic of the University Medical Center Freiburg. As of January 1, 2024, the Freiburg VHL Registry included 552 participants with data on ccRCC status. Patients lacking clinical data were excluded. In total, 375 (67.9%) patients had a VHL mutation classified as LOF1 or LOF2 by SGE (mean age ± SD = 45.5 years ± 17.6, 52% female). 122 participants had LOF1 mutations (age: 41.4 ± 14.3 years, 47.5% female). 253 participants had a LOF2 mutation (age: 47.5 ± 18.7 years, 54.2% female). 46 different LOF1 mutations and 11 different LOF2 mutations were present.<br><br>Other human-derived data used in analysis are publicly available, having been previously published and/or released by others, meaning no human participants were specifically recruited for this study. Sources of human genetic data analyzed in this study include ClinVar (https://www.ncbi.nlm.nih.gov/clinvar/), cBioPortal (https://www.cbioportal.org/), GeneBass (https://app.genebass.org/), TOPMed (https://bravo.sph.umich.edu/freeze8/hg38/) and VHLdb (http://vhldb.bio.unipd.it/). Sex and gender are not consistently reported in these database, precluding further analysis. |
| Reporting on race, ethnicity, or other socially relevant groupings | The Freiburg VHL Registry includes patients screened at least once until 2023 at the von Hippel-Lindau Outpatient Clinic of the University Medical Center Freiburg.<br><br>Other human-derived data used in analysis are publicly available, having been previously published and/or released by others, meaning no human participants were specifically recruited for this study. Sources of human genetic data analyzed in this study include ClinVar (https://www.ncbi.nlm.nih.gov/clinvar/), cBioPortal (https://www.cbioportal.org/), GeneBass (https://app.genebass.org/), TOPMed (https://bravo.sph.umich.edu/freeze8/hg38/) and VHLdb (http://vhldb.bio.unipd.it/). Race, ethnicity, and other socially relevant groupings are not consistently reported in these database, precluding further analysis. |
| Population characteristics | The Freiburg VHL Registry includes patients screened at least once until 2023 at the von Hippel-Lindau Outpatient Clinic of the University Medical Center Freiburg. As of January 1, 2024, the Freiburg VHL Registry included 552 participants with data on ccRCC status. Patients lacking clinical data were excluded. In total, 375 (67.9%) patients had a VHL mutation classified as LOF1 or LOF2 by SGE (mean age ± SD = 45.5 years ± 17.6, 52% female). 122 participants had LOF1 mutations (age: 41.4 ± 14.3 years, 47.5% female). 253 participants had a LOF2 mutation (age: 47.5 ± 18.7 years, 54.2% female). 46 different LOF1 mutations and 11 different LOF2 mutations were present.<br><br>Clinical data including age, gender and diagnostic results were recorded in a predefined database. |
| Recruitment | The Freiburg VHL Registry includes patients screened at least once until 2023 at the von Hippel-Lindau Outpatient Clinic of the University Medical Center Freiburg. All included patients have provided written informed consent. Inclusion criterion for this retrospective analysis was the detection of a VHL germline mutation assayed by SGE. Patients for whom no clinical data were available were excluded. |
| Ethics oversight | Use of the anonymised data for further analysis was approved by the ethics committee of Freiburg University (EK-FR 79/20). |

Note that full information on the approval of the study protocol must also be provided in the manuscript.

# Field-specific reporting

Please select the one below that is the best fit for your research. If you are not sure, read the appropriate sections before making your selection.

☒ Life sciences ☐ Behavioural & social sciences ☐ Ecological, evolutionary & environmental sciences

For a reference copy of the document with all sections, see nature.com/documents/nr-reporting-summary-flat.pdf

# Life sciences study design

All studies must disclose on these points even when the disclosure is negative.

| | |
|---|---|
| Sample size | We set out to study all possible single nucleotide variants across the coding sequence of VHL. This number is determined by multiplying the length of the DNA sequence being studied (in base pairs) by 3 (the number of possible single nucleotide variants at each position). In each analysis, all variants assayed falling into a specific category were included (e.g. all pathogenic or benign variants in ClinVar, all variants seen in a particular type of cancer). No specific sample sizes were chosen, but rather all qualifying variants successfully assayed in each category were included. The number of genetic variants present in each analysis are thus bounded by existent variation in the human population. |
| Data exclusions | A small fraction of experimental data were excluded on the basis of high experimental noise owing to low rates of CRISPR-mediated gene editing at certain genomic positions. These exclusions for technical reasons are discussed in the manuscript (see Results, Methods, and Supplementary Fig. 3). Such exclusions were not predetermined, but instead made using consistently applied thresholds designed to ensure data quality. ClinVar entries not meeting ClinVar's predetermined assertion criteria were excluded from analysis. |
| Replication | Two biological replicates (i.e. separate experiments from transfection forward) were performed and used to derive independent scores for each variant. Scores across replicates were well correlated (Fig. 2a). Where indicated, the average score for each variant across replicate experiments was used for analysis. |
| Randomization | One of many variants being assayed per experiment was introduced by chance to each cell via homologous recombination. Cells with different genetic variants were then treated the same, as part of a single pool of cells. For analysis, groups of variants were determined by patterns of genetic variation reported elsewhere (e.g. variants seen in cancer, variants seen in human germline testing, variants deemed pathogenic by clinicians). Therefore, randomization was not performed in this study. Covariates were not consistently reported across external human genetics databases analyzed, precluding their analysis. |
| Blinding | Experiments were internally controlled through the use of multiplexed assays, meaning variants being compared were all treated the same as part of a large pool of cells. In such instances, experimenters are inherently blind to which variants are present while carrying out assays, eliminating the need for blinding of individual samples. Blinding during analysis of SGE data was not performed, as all variants passing technical filters for data quality were treated identically without exclusions.<br><br>A final set of SGE data was used to retrospectively analyze clinical data, including pathogenicity assertions in ClinVar and cBioPortal. All analyses included all variants for which clinical data was available without exclusions. Clinical data was likewise analyzed in that all patients in a pre-defined cohort, the Freiburg VHL registry, were included for analysis without exclusion and analyzed retrospectively. |

# Reporting for specific materials, systems and methods

We require information from authors about some types of materials, experimental systems and methods used in many studies. Here, indicate whether each material, system or method listed is relevant to your study. If you are not sure if a list item applies to your research, read the appropriate section before selecting a response.

## Materials & experimental systems

| n/a | Involved in the study | |
|---|---|---|
| ☐ | ☒ | Antibodies |
| ☐ | ☒ | Eukaryotic cell lines |
| ☒ | ☐ | Palaeontology and archaeology |
| ☒ | ☐ | Animals and other organisms |
| ☐ | ☒ | Clinical data |
| ☒ | ☐ | Dual use research of concern |
| ☒ | ☐ | Plants |

## Methods

| n/a | Involved in the study | |
|---|---|---|
| ☒ | ☐ | ChIP-seq |
| ☐ | ☒ | Flow cytometry |
| ☒ | ☐ | MRI-based neuroimaging |

## Antibodies

| | |
|---|---|
| Antibodies used | Western blots: Mouse tubulin antibody (Sigma-Aldrich, T6199, 1:3,000), rabbit VHL antibody (Cell Signaling Technology, 68547, 1:1,1000), mouse HIF1A antibody (BD Transduction Laboratories, 610959, 1:1,000). Secondary antibodies: goat anti-mouse IgG-HRP (Abcam, ab205719, 1:10,000), goat anti-rabbit IgG-HRP (Sigma, AP307P, 1:10,000).<br><br>Immunofluorescence microscopy primary: rabbit anti-VHL (Cell Signaling Technologies, 68547, 1:200), mouse anti-HIF1α (Novus Biologicals, NB100-105, 1:50), |

| | |
|---|---|
| | Immunofluorescence secondary:<br>Donkey anti-Rabbit IgG Alexa Fluor 555 (Thermo Fisher, A-31572, 1:500), goat anti-Mouse IgG Alexa Fluor 647 (Thermo Fisher, A-21235, 1:500) |
| Validation | All antibodies used are well-validated for use in the indicated applications in human cells, as evidenced by a wealth of citations and validation statements and data described on the manufacturer websites:<br><br>Mouse tubulin antibody (Sigma-Aldrich, T6199) is a Sigma "enhanced validation" antibody with at least 10 references (https://www.sigmaaldrich.com/GB/en/product/sigma/t6199?icid=sharepdp-clipboard-copy-productdetailpage).<br><br>Rabbit VHL antibody (Cell Signaling Technology, 68547) has 95 citations (https://www.cellsignal.com/products/primary-antibodies/vhl-antibody/68547).<br><br>Mouse HIF1A antibody (BD Transduction Laboratories, 610959) has 7 citations. (https://www.bdbiosciences.com/en-gb/products/reagents/microscopy-imaging-reagents/immunofluorescence-reagents/purified-mouse-anti-human-hif-1.610959)<br><br>Mouse anti-HIF1α (Novus Biologicals, NB100-105) has been cited over 1,000 times: https://www.novusbio.com/products/hif-1-alpha-antibody-h1alpha67_nb100-105#datasheet.<br><br>In data provided in the manuscript, specificity of all primary antibodies for HIF1A and VHL was confirmed via genetic manipulation of HAP1 cells (i.e. VHL knock-out), leading to the expected change in staining by both western blot and immunofluorescence (EDF6).<br><br>Secondary antibodies used for immunofluorescence microscopy were assessed for background staining using both knockout lines lacking VHL and staining controls lacking primary antibody. |

# Eukaryotic cell lines

Policy information about cell lines and Sex and Gender in Research

| | |
|---|---|
| Cell line source(s) | The parental HAP1 cell line was originally obtained from the commercial supplier formerly known as Haplogen (now Horizon Discovery). HAP1 cells were derived from a male human but are haploid and lack a Y chromosome. |
| Authentication | The HAP1 cells used in this study were commercially sourced and were not independently authenticated. Sanger sequencing was performed on clonal populations where indicated to confirm genetic manipulations (i.e. knock-out generation). |
| Mycoplasma contamination | All cell lines used in this study tested negative for mycoplasma contamination. |
| Commonly misidentified lines<br>(See ICLAC register) | None used. |

# Clinical data

Policy information about clinical studies

All manuscripts should comply with the ICMJE guidelines for publication of clinical research and a completed CONSORT checklist must be included with all submissions.

| | |
|---|---|
| Clinical trial registration | n/a |
| Study protocol | This was a retropective cohort analysis without a study protocol |
| Data collection | The Freiburg VHL Registry includes patients screened at least once through 2023 at the von Hippel-Lindau Outpatient Clinic of the University Medical Center Freiburg. Clinical data such as age, gender and diagnostic results were recorded in a predefined database. Clinical surveillance was performed according to international guidelines for VHL disease (VHL Active Surveillance Guidelines) and included an MRI and/or CT scan of the abdomen for the diagnosis of ccRCC. |
| Outcomes | For ccRCC, the first radiologic description was considered the initial diagnosis of ccRCC. Registrants without ccRCC were censored at the age of their last visit. |

# Flow Cytometry

## Plots

Confirm that:

☒ The axis labels state the marker and fluorochrome used (e.g. CD4-FITC).

☒ The axis scales are clearly visible. Include numbers along axes only for bottom left plot of group (a 'group' is an analysis of identical markers).

☒ All plots are contour plots with outliers or pseudocolor plots.

☒ A numerical value for number of cells or percentage (with statistics) is provided.

## Methodology

| | |
|---|---|
| Sample preparation | pSCR plasmids containing sequences of interest between EGFP and mCherry genes were cloned (see Methods). Each vector was transfected into HAP1-LIG4KO cells as described. On day 5 post-transfection, cells were trypsinized, washed, and resuspended in FACS buffer. |
| Instrument | BD Fortessa X20 flow cytometer |
| Software | FlowJo v10.10 |
| Cell population abundance | Data was recorded for at least 150,000 single cells per sample. |
| Gating strategy | Quadrant gating was performed (FlowJo) to determine the fraction of transfected cells (EGFP+) that were mCherry+. Prior gating on living, single cells was performed using FSC/SSC. |

☒ Tick this box to confirm that a figure exemplifying the gating strategy is provided in the Supplementary Information.
