# Supplementary material for: Saturation genome editing maps the functional spectrum of pathogenic VHL alleles
Source: Nat Genet. 2024 Jul 5;56(7):1446–55. doi: 10.1038/s41588-024-01800-z (PMC11250436; doi:10.1038/s41588-024-01800-z)

Extended Data Fig. 6a unprocessed scans

Extended Data Fig. 6b unprocessed scans

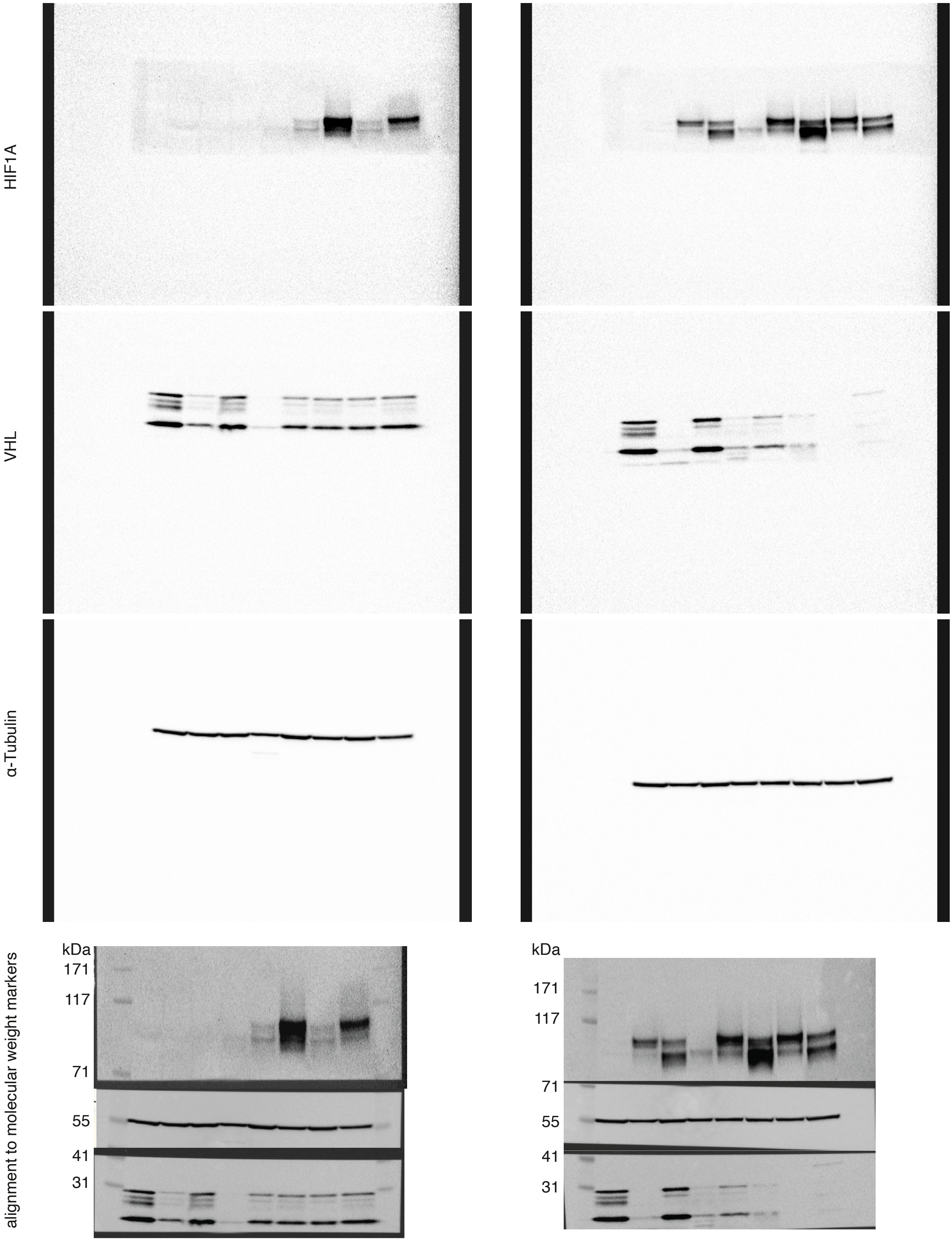

Supplement: Supplementary file 6 — Unprocessed western blot images. [file 41588_2024_1800_MOESM6_ESM.pdf]
